# Association of Post-Neoadjuvant Chemotherapy MRI and ^18^F-FDG PET/CT Findings with Tumor Response and Prognosis in Breast Cancer

**DOI:** 10.3390/diagnostics16050713

**Published:** 2026-02-27

**Authors:** Burçin Çakan Demirel, Semra Taş, Ayberk Bayramgil, Anıl Yıldız, Şahin Bedir, Nigar Erkoç, Aynur Özen, Merve Tokoçin, Nida Sünnetçi Arıkan, Ali Muhammedoğlu, Yunus Emre Altıntaş, Ahmet Bilici

**Affiliations:** 1 Department of Medical Oncology, School of Medicine, Istanbul Medipol University, Istanbul Medipol Mega University Hospital, 34214 Istanbul, Türkiye; abilici@medipol.edu.tr; 2 Department of Medical Oncology, School of Medicine, Pamukkale University, 20160 Denizli, Türkiye; 3 Department of Medical Oncology, Umraniye Training and Research Hospital, 34764 Istanbul, Türkiye; 4 Department of Medical Oncology, Basaksehir Cam ve Sakura City Hospital, 34480 Istanbul, Türkiye; anilyildiz@live.com; 5 Department of Medical Oncology, Istinye University, Gaziosmanpaşa Hastanesi, 34240 Istanbul, Türkiye; 6 Department of Radiology, Bağcılar Training and Research Hospital, 34164 Istanbul, Türkiye; 7 Department of Nuclear Medicine, Bağcılar Training and Research Hospital, 34164 Istanbul, Türkiye; 8 Department of General Surgery, Bağcılar Training and Research Hospital, 34164 Istanbul, Türkiye; 9 Department of Radiation Oncology, Bağcılar Training and Research Hospital, 34164 Istanbul, Türkiye; 10 Department of Pathology, Bağcılar Training and Research Hospital, 34164 Istanbul, Türkiye

**Keywords:** breast cancer, neoadjuvant chemotherapy, pathological complete response, magnetic resonance imaging, ^18^F-fluorodeoxyglucose positron emission tomography/computed tomography, molecular subtypes, survival outcomes

## Abstract

Accurate non-invasive prediction of pathological complete response (pCR) following neoadjuvant chemotherapy (NACT) in breast cancer (BC) remains challenging despite its established prognostic significance. **Objective**: We aimed to evaluate the prognostic utility of baseline and post-NACT magnetic resonance imaging (MRI) and ^18^F-fluorodeoxyglucose positron emission tomography/computed tomography (^18^F-FDG PET/CT) for predicting pCR and survival outcomes, focusing on molecular subtype-specific performance and post-NACT imaging discordance. **Methods**: In this multicenter study, we retrospectively analyzed 335 patients with BC who received NACT between 2015 and 2025. Baseline (pre-NACT) and post-NACT imaging assessments were performed using MRI and ^18^F-FDG PET/CT. Pathological response was graded using the Miller–Payne classification system. Multivariable logistic regression was applied to identify independent predictors of pCR, whereas survival outcomes were examined using Kaplan–Meier analysis and Cox regression. **Results**: The overall pCR rate was 41.2%. Post-NACT imaging demonstrated complete response in 58.7% of patients by ^18^F-FDG PET/CT and 43.6% by MRI, both significantly correlating with pCR (*p* < 0.001). Pre-NACT MRI tumor size showed predictive value exclusively in Luminal A/B HER2-negative disease (area under curve = 0.681; *p* = 0.013). Importantly, post-NACT discordance between MRI and ^18^F-FDG PET/CT-based tumor size assessments was an independent predictor of both mortality (hazard ratio, 1.03) and disease progression (hazard ratio, 1.01). **Conclusions**: Post-NACT MRI and ^18^F-FDG PET/CT findings correlate strongly with pCR achievement, whereas pre-NACT MRI tumor size predicts pCR only in hormone receptor-positive HER2-negative subtypes. Importantly, post-NACT imaging discordance independently predicted mortality and disease progression, suggesting that dual-modality imaging assessment may identify high-risk patients requiring intensified surveillance.

## 1. Introduction

Neoadjuvant chemotherapy (NACT) has become standard treatment for locally advanced breast cancer (BC) and is increasingly utilized in early-stage disease to facilitate breast-conserving surgery and provide early prognostic information [1]. The achievement of pathological complete response (pCR) following NACT serves as a validated surrogate endpoint for long-term survival and has become a critical determinant of treatment efficacy [2,3]. Consequently, accurate non-invasive assessment of response to NACT is essential to guide therapeutic decisions and potentially identify patients who may benefit from treatment modification or intensified surveillance [4].

Magnetic resonance imaging (MRI) and ^18^F-fluorodeoxyglucose positron emission tomography/computed tomography (^18^F-FDG PET/CT) represent the principal advanced imaging modalities for evaluating NACT response in patients with BC [5,6,7]. While breast MRI provides superior soft tissue contrast and morphological detail for measuring residual tumor burden [8], ^18^F-FDG PET/CT offers functional metabolic information reflecting cancer cell viability [9]. However, the comparative accuracy of these modalities in predicting pCR remains debated [4,5,6,7]. This uncertainty is particularly relevant given the marked biological heterogeneity of BC.

In this regard, molecular subtypes defined by hormone receptor (HR) and human epidermal growth factor receptor 2 (HER2) status are characterized by substantially heterogeneous responses to NACT, with HER2-positive and triple-negative subtypes achieving higher pCR rates than HR-positive/HER2-negative disease [10,11]. This heterogeneity raises fundamental questions regarding whether imaging modalities demonstrate equivalent predictive performance across all subtypes, or whether subtype-specific imaging strategies are warranted. Beyond response prediction, the prognostic implications of discordance between MRI and ^18^F-FDG PET/CT imaging findings remain incompletely characterized. Accordingly, understanding the relationship between post-NACT imaging discrepancies, molecular subtype, and survival outcomes could identify patients at increased risk of treatment failure despite apparently favorable imaging findings.

To address these knowledge gaps, we conducted a multicenter comparative evaluation of MRI and ^18^F-FDG PET/CT in a large, molecularly characterized cohort of patients with BC receiving contemporary NACT regimens, focusing on subtype-specific predictive performance and the prognostic significance of post-NACT imaging discordance.

## 2. Materials and Methods

### 2.1. Study Design and Patient Population

This retrospective, multicenter cohort study enrolled consecutive patients treated at three tertiary referral centers in Istanbul, Türkiye—Bağcılar Training and Research Hospital, Medipol Mega University Hospital, and Yeni Yüzyıl University Gaziosmanpaşa Hospital—between January 2015 and January 2025.

Eligible patients were adults (aged ≥18 years) with histopathologically confirmed invasive breast cancer established by core needle biopsy who had received a complete neoadjuvant chemotherapy (NACT) regimen. All participants were required to have undergone both baseline (pre-NACT) and post-NACT breast MRI as well as baseline and post-NACT ^18^F-FDG PET/CT, with each imaging modality performed at the same center at both time points to minimize inter-scanner variability. Only patients who subsequently proceeded to definitive breast surgery with complete pathological evaluation were retained in the final cohort. Patients were excluded if they did not undergo surgical resection, had incomplete imaging or insufficient follow-up data, or presented with synchronous malignancies.

The study protocol was approved by the Bağcılar Training and Research Hospital Clinical Research Ethics Committee (approval date: 3 June 2025). The investigation was conducted in accordance with the principles of the Declaration of Helsinki; informed consent was waived owing to the retrospective design and the use of de-identified data.

### 2.2. Imaging Protocols and Response Assessment

^18^F-FDG PET/CT and breast MRI were performed at two time points: baseline staging and post-NACT response assessment. All imaging studies were conducted as part of routine clinical practice at the participating centers. To ensure imaging standardization, only patients whose baseline and post-NACT ^18^F-FDG PET/CT examinations were performed at the same center and whose baseline and post-NACT breast MRI examinations were conducted at the same center were included in the study. This approach was adopted to minimize inter-scanner variability and maintain consistency in acquisition protocols and reconstruction algorithms between baseline and post-NACT examinations.

Breast MRI was performed at baseline and post-NACT using 1.5-T or 3-T systems with dedicated breast coils. Imaging protocols included T1-weighted, T2-weighted fat-suppressed, diffusion-weighted, and DCE sequences. DCE-MRI was acquired following intravenous administration of gadolinium-based contrast material (0.1 mmol/kg). Tumor size was defined as the longest diameter of the enhancing invasive tumor on DCE-MRI and was measured on the post-contrast phase in which tumor enhancement was most clearly delineated. In cases of non-mass enhancement or multifocal disease, the largest contiguous enhancing lesion was measured according to RECIST 1.1 criteria. Absence of residual enhancement at the tumor bed was considered imaging complete response. MRI-based treatment response was categorized as complete response, partial response, stable disease, or progressive disease based on changes in tumor size between baseline and post-NACT examinations [12]. MRI evaluations were performed by experienced breast radiologists blinded to pathological outcomes, with consensus readings used in cases of discrepancy.

For ^18^F-FDG PET/CT imaging, patients fasted for at least 6 h prior to radiotracer administration, and blood glucose levels were confirmed to be within acceptable clinical limits before injection. ^18^F-FDG was administered intravenously at a weight-based dose (approximately 3.7–5.2 MBq/kg). Image acquisition was performed approximately 60 min after tracer injection. Low-dose CT was acquired for attenuation correction and anatomical localization, followed by three-dimensional PET emission imaging. PET images were reconstructed using an ordered-subset expectation maximization (OSEM) algorithm in accordance with institutional protocols. Given the retrospective, multicenter design, specific reconstruction parameters—including time per bed position, matrix size, and number of OSEM iterations and subsets—varied across centers and over the study period in accordance with institutional protocols and scanner specifications. Inter-scanner variability was mitigated by requiring that each patient’s baseline and post-NACT ^18^F-FDG PET/CT examinations were performed at the same center, ensuring consistency of acquisition and reconstruction parameters between the two time points for each individual patient.

Breast cancer metabolic activity was quantitatively assessed using SUVmax, defined as the highest ^18^F-FDG uptake within a single voxel of the primary tumor [13]. Baseline and post-NACT SUVmax values were recorded, and metabolic change (ΔFDG) was calculated as the difference between these values [13]. Tumor size measurements on ^18^F-FDG PET/CT were obtained from the CT component of the PET/CT examination using the longest axial diameter of the primary lesion. PET images were used to assist in lesion localization when anatomical delineation was challenging.

Metabolic treatment response on ^18^F-FDG PET/CT was categorized as complete response (absence of abnormal FDG uptake at the primary tumor site, comparable to surrounding background tissue), partial response (a clear reduction in FDG uptake compared with baseline, as assessed visually and semi-quantitatively), stable disease (minimal change in uptake), or progressive disease (increase in FDG uptake or appearance of new hypermetabolic lesions), based on relative changes in FDG uptake between baseline and post-NACT examinations [14]. Due to the retrospective design, strict PERCIST criteria could not be uniformly applied across all centers. Post-NACT MRI-PET discordance was defined as the absolute difference between post-NACT tumor sizes measured by MRI and ^18^F-FDG PET/CT.

It should be noted that tumor size on MRI was measured as the longest diameter of enhancing tumor on DCE sequences, whereas tumor size on ^18^F-FDG PET/CT was derived from the CT component using the longest axial diameter. Because these two measurements rely on fundamentally different tissue contrast mechanisms—contrast enhancement versus anatomical attenuation—some degree of inter-modality measurement variability is inherent. To mitigate this, all measurements were performed by experienced readers using standardized criteria at each center, and the requirement for same-center baseline and post-NACT imaging ensured consistency of acquisition parameters within each modality. The discordance metric was therefore designed to capture biologically meaningful differences in residual disease assessment rather than random measurement noise.

### 2.3. Pathological Assessment and Molecular Subtyping

Pretreatment core needle biopsy specimens were reviewed to extract baseline tumor characteristics, including histological subtype, hormone receptor status (estrogen receptor [ER] and progesterone receptor [PR]), human epidermal growth factor receptor 2 (HER2) status (determined by immunohistochemistry and/or fluorescence in situ hybridization), and Ki-67 proliferation index. Estrogen receptor status was classified as positive (>10% nuclear staining by immunohistochemistry), weakly positive (1–10% nuclear staining), or negative (<1% staining). The “weakly positive” category corresponds to the increasingly recognized “ER-low” phenotype, which exhibits biological and therapeutic response characteristics that may differ from those of strongly ER-positive tumors. For the purposes of molecular subtyping, tumors with weakly positive ER (1–10%) were grouped with ER-positive tumors, consistent with clinical practice during the study period. Molecular BC subtypes were classified as follows: Luminal A (ER+/HER2−/Ki-67 < 20%), Luminal B HER2-negative (ER+/HER2−/Ki-67 ≥ 20%), Luminal B HER2-positive (ER+/HER2+), HER2-enriched (ER−/HER2+), and triple-negative (ER−/PR−/HER2−) [15]. Following surgical resection, a comprehensive pathological evaluation was performed to assess residual disease burden, including the presence and extent of residual invasive tumor, postoperative tumor size, lymph node status, and residual ductal carcinoma in situ.

### 2.4. Pathological Response to NACT

Pathological response to NACT was assessed using the Miller–Payne classification (grades 1–5), which compares tumor cellularity in the post-treatment surgical specimen with that in the pretreatment core biopsy [16]. Grade 1 indicates no reduction in tumor cells, grade 2 a minor reduction (<30%), grade 3 a moderate reduction (approximately 30–90%), and grade 4 a marked reduction with >90% loss of invasive tumor cells. Grade 5 corresponds to pCR, defined as the absence of residual invasive carcinoma in the breast, irrespective of any residual ductal carcinoma in situ.

### 2.5. Adjuvant Treatment

Following definitive surgery, adjuvant treatment was administered in accordance with institutional protocols and national guidelines. Adjuvant radiotherapy was delivered to all patients who underwent breast-conserving surgery and was also administered to patients who underwent mastectomy when indicated by tumor stage, nodal involvement, or other high-risk features. Patients with hormone receptor-positive disease received adjuvant endocrine therapy (tamoxifen or aromatase inhibitors, depending on menopausal status) for a planned duration of at least 5 years. Patients with HER2-positive tumors who had not completed 1 year of anti-HER2 therapy in the neoadjuvant setting continued trastuzumab (±pertuzumab) in the adjuvant setting. For patients with residual invasive disease after NACT, adjuvant treatment escalation was considered on an individual basis: patients with HER2-positive residual disease were offered trastuzumab emtansine (T-DM1), and those with triple-negative residual disease were considered for adjuvant capecitabine. Detailed data on specific adjuvant systemic regimens were not systematically collected owing to the retrospective design.

### 2.6. Survival Outcomes

Overall survival (OS) was measured from diagnosis until death from any cause or the last follow-up (10 May 2025). Progression-free survival (PFS) was defined as the interval between diagnosis and disease progression, death, or the last follow-up (10 May 2025).

### 2.7. Statistical Analysis

Continuous variables were assessed for normality using the Kolmogorov–Smirnov test and visual inspection of histograms. Normally distributed variables were expressed as mean ± standard deviation (SD), whereas non-normally distributed variables were summarized as median (minimum–maximum), when appropriate. Categorical variables were expressed as numbers (*n*) and percentages (%). Comparisons between groups were performed using Student’s independent-sample *t*-test for normally distributed continuous data between two groups, one-way analysis of variance (ANOVA) with Bonferroni post hoc correction for comparisons across three or more groups, Pearson’s chi-square test for categorical variables with adequate expected cell frequencies, or Fisher’s exact test when expected cell counts fell below five, as appropriate according to data distribution and variable type. The choice of parametric versus non-parametric tests was guided by the results of the normality assessment described above. To identify independent predictors of pCR, variables with *p* < 0.10 in univariable analysis were entered into a multivariable logistic regression model using the enter method to control for potential confounding**; this threshold was selected to minimize the risk of excluding potentially relevant covariates from the multivariable model. Results were expressed as odds ratios (ORs) with 95% confidence intervals (CIs). The discriminative ability of imaging findings for pCR prediction across molecular subtypes was assessed using receiver operating characteristic (ROC) curve analysis. ROC analysis was chosen because it provides a threshold-independent measure of a test’s ability to discriminate between two states (pCR versus non-pCR) and is widely recommended for evaluating diagnostic and predictive biomarkers in oncology. Area under the curve (AUC) values with 95% CIs were calculated, and optimal cut-off points were determined using the Youden index, which maximizes the sum of sensitivity and specificity and thereby identifies the threshold that best balances diagnostic trade-offs. Survival analysis was conducted using Kaplan–Meier methods, which allow estimation of time-to-event probabilities while accounting for censored observations, with log-rank tests used to compare survival curves between groups. Univariable and multivariable Cox proportional hazards regression models were constructed to identify factors associated with mortality and disease progression. The proportional hazards assumption was verified through log-minus-log plots and Schoenfeld residuals; no significant violations were identified. Results were given as hazard ratios (HRs) with 95% CIs. To minimize the risk of overfitting, the events-per-variable (EPV) ratio was assessed in both multivariable Cox regression models. For overall survival, a total of 41 death events occurred, and two covariates were included in the final multivariable model, resulting in an EPV of 20.5. For progression-free survival, 42 progression events occurred, and four covariates were included (pCR status, MRI-PET discordance, ΔFDG, and age group), yielding an EPV of 10.5. Both values exceed the recommended minimum threshold of 10 events per variable, providing reassurance against overfitting in the final models. Statistical analyses were performed using IBM SPSS Statistics Version 25.0 (IBM Corp., Armonk, NY, USA). Two-tailed *p*-values < 0.05 were considered statistically significant.

## 3. Results

### 3.1. Patient Characteristics

A total of 335 patients with biopsy-confirmed invasive BC who received NACT were included in the study (Table 1). Most participants presented with clinical stage II disease (62.4%) and T2 tumors (66.0%). The predominant molecular subtype was Luminal B HER2-positive (40.6%), followed by Luminal B HER2-negative (22.1%), HER2-enriched (18.5%), triple-negative (11.9%), and Luminal A (6.9%). The most common NACT regimens were 4AC–taxane–trastuzumab–pertuzumab (40.6%) and 4AC–taxane (36.1%), with a mean of 11.6 ± 4.5 chemotherapy cycles administered. The overall pCR rate was 41.2% (138/335). Over a median follow-up of 34.9 months (range, 0.33–125.43), disease progression and mortality occurred in 12.5% (*n* = 42) and 12.2% (*n* = 41) of patients, respectively.

### 3.2. Post-NACT Imaging Findings by Miller–Payne Pathological Grade

The distribution of post-NACT imaging findings according to Miller–Payne pathological grade is reported in Table 2. Complete radiological response was observed in 197 of 335 patients (58.7%) on ^18^F-FDG PET/CT and in 146 of 335 patients (43.6%) on MRI. Both imaging modalities showed strong correlation with the achievement of pCR as assessed by the Miller–Payne grading system (both *p* < 0.001). It is important to note that “imaging complete response” and pCR reflect different constructs: the former indicates absence of detectable disease on a given imaging modality, whereas the latter requires histopathological confirmation of no residual invasive carcinoma. The overall pCR rate was 41.2%, while imaging complete response rates were 43.6% for MRI and 58.7% for ^18^F-FDG PET/CT. The closer approximation of the MRI complete response rate (43.6%) to the true pCR rate (41.2%) suggests that MRI may be more specific in identifying true complete responders, whereas ^18^F-FDG PET/CT—despite its higher sensitivity for detecting metabolic quiescence—classified an additional 17.5% of patients as complete responders who did not achieve pCR at pathological examination, indicating a higher false-positive rate for this modality.

Among the 138 patients achieving pCR, post-NACT complete response was identified in 115 patients (83.3%) by ^18^F-FDG PET/CT and in 110 patients (79.7%) by MRI. Conversely, among the 20 patients with Miller–Payne grade 1 (minimal or no response), no complete responses were detected by ^18^F-FDG PET/CT, whereas MRI identified complete response in only 1 patient (5.0%). Post-NACT ^18^F-FDG PET/CT SUVmax demonstrated a progressive decline across increasing Miller–Payne grades, with values of 6.15 ± 5.89 in grade 1 patients compared to 0.45 ± 1.33 in grade 5/pCR patients (*p* < 0.001). Similarly, ΔFDG increased progressively from 3.85 ± 6.42 in grade 1 to 9.67 ± 5.50 in grade 5 (*p* < 0.001). Post-NACT tumor size measurements by both MRI and ^18^F-FDG PET/CT also showed a significant correlation with Miller–Payne grades (both *p* < 0.001). When pCR and non-pCR patients were compared directly, post-NACT ^18^F-FDG PET/CT SUVmax was significantly lower in pCR patients (0.45 ± 1.33 versus 2.83 ± 5.12; *p* < 0.001), while metabolic change (ΔFDG) was significantly greater in pCR patients (9.67 ± 5.50 versus 7.51 ± 6.73; *p* = 0.002).

### 3.3. Predictors of pCR

On univariable analysis, patients who achieved pCR had smaller baseline tumors on both MRI (30.87 ± 14.87 mm versus 34.15 ± 16.14 mm; *p* = 0.049) and ^18^F-FDG PET/CT (29.20 ± 15.59 mm versus 32.65 ± 16.30 mm; *p* = 0.048). Additional variables associated with pCR at a threshold of *p* < 0.10 in univariable analysis included clinical stage, ER status, weakly positive HER2 status, negative HER2 status, Ki-67, ΔFDG, the absence of a residual in situ component, and the number of chemotherapy cycles. In multivariable logistic regression adjusting for potential confounders, only weakly positive ER status (OR = 0.22; 95% CI, 0.10–0.46; *p* < 0.001), negative ER status (OR = 0.38; 95% CI, 0.15–0.93; *p* = 0.035), and the absence of a residual in situ component (OR = 2.70; 95% CI, 1.57–4.64; *p* < 0.001) remained independently associated with pCR, whereas baseline MRI and ^18^F-FDG PET/CT findings did not. The finding that weakly positive (ER-low, 1–10%) and negative ER status were both inversely associated with pCR likely reflects the lower chemosensitivity of ER-expressing tumors compared with ER-negative subtypes, particularly in a cohort with a high proportion of HER2-positive disease where anti-HER2 targeted therapy drives pCR irrespective of ER status.

### 3.4. Subtype-Specific Performance of Baseline Imaging Findings in Predicting pCR

ROC curve analysis of baseline imaging parameters revealed considerable variability in their ability to predict pCR across BC molecular subtypes (Table 3). In the hormone receptor-positive, HER2-negative subgroup (Luminal A and Luminal B HER2-negative; *n* = 97), baseline MRI tumor size showed significant discriminative performance for predicting pCR (AUC, 0.681; 95% CI, 0.558–0.804; *p* = 0.013). An optimal cut-off value of ≤28.5 mm yielded 60.0% sensitivity and 62.2% specificity. In the same subgroup, baseline ^18^F-FDG PET/CT tumor size demonstrated modest but statistically significant predictive value (AUC, 0.654; 95% CI, 0.527–0.781; *p* = 0.039), with an optimal cut-off of ≤26.0 mm providing 57.9% sensitivity and 56.8% specificity. Conversely, among patients with biologically aggressive subtypes (Luminal B HER2-positive, HER2-enriched, and triple-negative; *n* = 238), none of the baseline imaging metrics showed statistically significant discriminative ability for pCR prediction.

### 3.5. Survival Outcomes and Prognostic Factors

**Figure 1:**
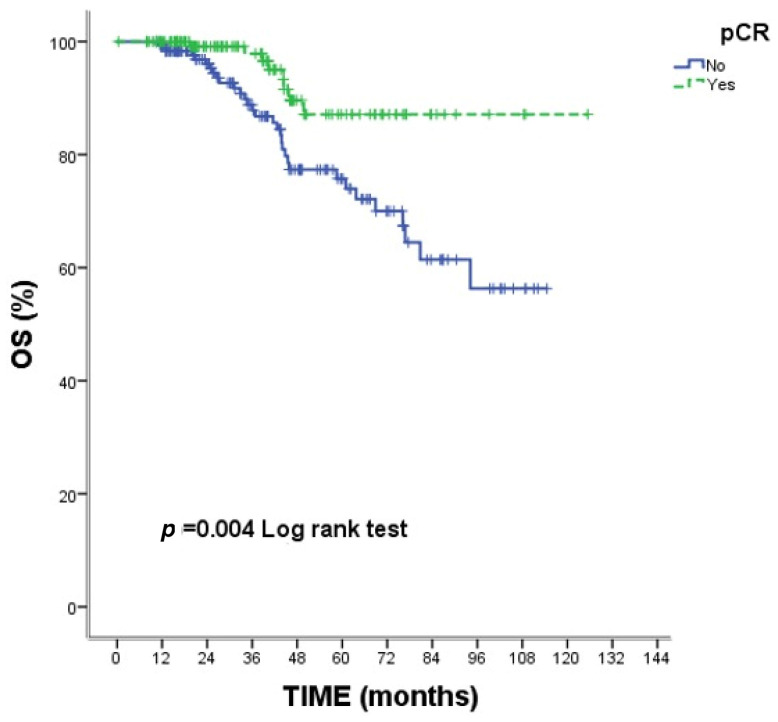
Kaplan–Meier curve for overall survival (OS) according to pathological complete response (pCR) status. Patients achieving pCR demonstrated significantly improved overall survival compared with those without pCR (log-rank *p* = 0.004).

**Figure 2:**
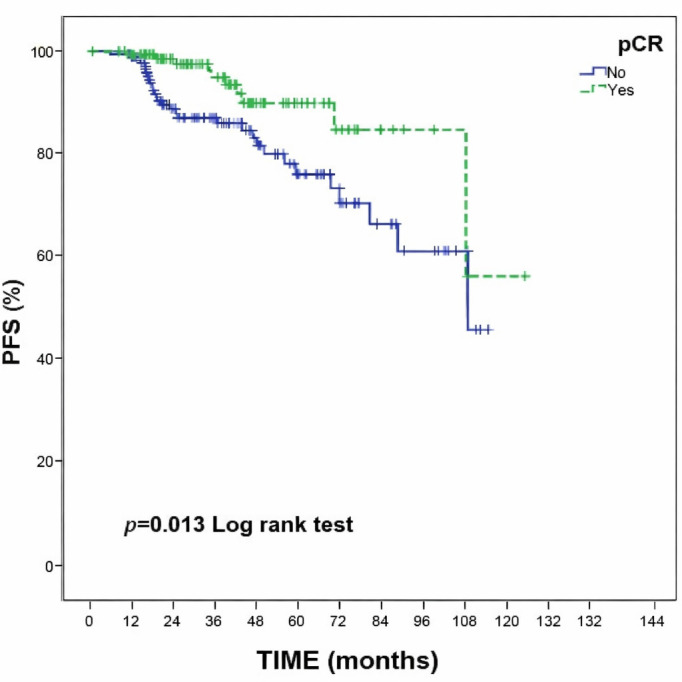
Kaplan–Meier curve for progression-free survival (PFS) according to pathological complete response (pCR) status. Patients achieving pCR demonstrated significantly improved progression-free survival compared with those without pCR (log-rank *p* = 0.013).

At a median follow-up of 34.9 months, patients who achieved pCR exhibited significantly improved survival outcomes compared with those without pCR. The estimated 5-year OS was 87.1% in the pCR group and 75.7% in the non-pCR group (log-rank *p* = 0.004). Likewise, the 5-year PFS was 89.9% and 76.2%, respectively (log-rank *p* = 0.013). Kaplan–Meier plots for OS and PFS according to pCR status are shown in Figure 1 and Figure 2.

In multivariable Cox regression models of OS and PFS (Table 4), pCR independently predicted a reduced risk of mortality (HR = 0.34; 95% CI, 0.10–0.94; *p* = 0.008) and disease progression (HR = 0.40; 95% CI, 0.19–0.86; *p* = 0.019) after adjustment for potential confounders. Notably, post-NACT MRI-PET discordance emerged as an independent adverse prognostic factor. Each 1-mm increase in the absolute difference between post-NACT MRI and ^18^F-FDG PET/CT tumor size measurements was independently associated with a 3% increase in mortality risk (HR = 1.03; 95% CI, 1.01–1.15; *p* = 0.005) and a 1% increase in progression risk (HR = 1.01; 95% CI, 1.00–1.03; *p* = 0.049).

## 4. Discussion

This multicenter study of 335 patients with BC receiving NACT yielded three principal findings with implications for response assessment and risk stratification. First, post-NACT MRI and ^18^F-FDG PET/CT demonstrated robust correlation with pCR, whereas baseline imaging parameters predicted pCR exclusively in hormone receptor-positive HER2-negative disease. Second, molecular subtype was found to significantly affect the predictive utility of baseline anatomic imaging. Third, discordance between post-NACT MRI and ^18^F-FDG PET/CT tumor size measurements independently predicted adverse survival outcomes, suggesting that dual-modality assessment identifies high-risk patients despite apparently favorable pathological response.

The overall pCR rate of 41.2% observed in our study is in accordance with contemporary series incorporating dual HER2-targeted therapy [17,18] and reflects our cohort’s predominance of biologically aggressive subtypes (59.4% HER2-positive, 11.9% triple-negative). Notably, we found that post-NACT imaging demonstrated complete response in 58.7% of patients by ^18^F-FDG PET/CT and 43.6% by MRI, both correlating strongly with Miller–Payne pathological grades. The higher rate of imaging complete response on ^18^F-FDG PET/CT relative to MRI (58.7% versus 43.6%)—and, critically, relative to the true pCR rate (41.2%)—indicates that metabolic imaging, while more sensitive for detecting treatment response, carries a higher false-positive rate for predicting pCR. In practical terms, approximately 17% of patients were classified as complete metabolic responders on PET/CT despite harboring residual invasive disease at pathological examination. Conversely, the MRI complete response rate (43.6%) more closely approximated the true pCR rate, suggesting greater specificity of anatomic imaging for this endpoint. This complementary performance profile—higher sensitivity for PET/CT, higher specificity for MRI—provides a rationale for the dual-modality approach employed in this study, as neither modality alone captures the full spectrum of treatment response.

These findings confirm and expand prior investigations establishing metabolic imaging’s superior sensitivity for detecting residual viable tumor [5,6]. In a recent study, AlBuainain et al. [5] reported ^18^F-FDG PET/CT accuracy of 79.4% versus 70.5% for MRI in predicting pCR, with ^18^F-FDG PET/CT demonstrating superior negative predictive value (94.4% versus 78.2%). Our results are broadly consistent with these estimates, reinforcing the emerging consensus that metabolic imaging outperforms anatomic assessment for post-NACT response evaluation. Despite this strong correlation, the observed false-negative rates observed in this study merit consideration. Among 138 patients achieving pCR, 16.7% demonstrated residual metabolic activity on ^18^F-FDG PET/CT and 20.3% showed residual enhancement on MRI. These rates are consistent with published estimates [19,20] and may reflect physiological imaging–pathology discordance. Specifically, residual ^18^F-FDG uptake without viable tumor may represent inflammatory infiltration or reactive fibrosis following chemotherapy-induced necrosis [21]. Similarly, persistent MRI enhancement may reflect vascular permeability in treatment-induced fibrosis or ductal carcinoma in situ [22], particularly in hormone receptor-positive disease where microscopic foci challenge anatomic resolution. Beyond post-NACT assessment, a central finding of this investigation is the marked heterogeneity in baseline imaging predictive performance across different BC molecular subtypes. In hormone receptor-positive HER2-negative disease, baseline MRI tumor size demonstrated significant discriminative ability for pCR prediction, yielding 60.0% sensitivity and 62.2% specificity. Baseline ^18^F-FDG PET/CT tumor size also showed comparable performance. Conversely, among patients with biologically aggressive subtypes (*n* = 238), none of the baseline imaging measurements exceeded chance discrimination (MRI AUC = 0.528, *p* = 0.471; ^18^F-FDG PET/CT AUC = 0.565, *p* = 0.091). This divergence reflects fundamental differences in BC biology and treatment response mechanisms. Hormone receptor-positive HER2-negative tumors exhibit substantial biological heterogeneity, with tumor burden and proliferative activity serving as independent determinants of chemotherapy sensitivity [23]. The biological basis for size-response associations in luminal disease likely reflects clonal heterogeneity and increased probability of chemotherapy-resistant subclones in larger tumor masses [24]. Conversely, HER2-positive and triple-negative tumors demonstrate inherently high proliferative activity [25] that may supersede tumor volume as the dominant determinant of pCR. Recent multiomic profiling by Mo et al. [26] identified distinct molecular patterns predicting pCR in a subtype-specific manner, with tumor size contributing minimally to predictive models for HER2-positive and triple-negative disease. These findings carry important methodological implications for imaging biomarker development, as subtype-agnostic analyses may obscure biologically meaningful associations, leading to suboptimal conclusions about imaging utility. Taken together, these observations suggest that the role of baseline imaging in predicting treatment response should be contextualized within the molecular landscape of the individual tumor, rather than applied uniformly across all breast cancer subtypes.

One of the most intriguing findings in our study is the identification of post-NACT MRI-PET discordance as an independent predictor of mortality (HR = 1.03 per mm; *p* = 0.005) and disease progression (HR = 1.01 per mm; *p* = 0.049), independent of pCR achievement. The prognostic significance of multimodality imaging discordance represents a conceptually novel observation with limited precedent. Several biological mechanisms may explain this finding. First, discordance may indicate spatial heterogeneity in treatment response, with metabolically active residual tumor distributed throughout treatment-induced fibrosis, creating functional–anatomic mismatch [27]. Tumors exhibiting such spatial discordance may therefore harbor microenvironmental niches supporting chemotherapy-resistant clones. Second, discordance may reflect differential assessment of residual disease components, as MRI enhancement can persist in ductal carcinoma in situ [28], whereas ^18^F-FDG PET/CT preferentially identifies viable invasive carcinoma [29]. Third, imaging discordance may serve as a surrogate for aggressive tumor biology. The observation that discordance retained prognostic significance after adjusting for pCR status supports this interpretation, suggesting that biological determinants of imaging–pathology mismatch confer risk beyond that attributable to residual invasive carcinoma at pathological examination. With regard to the practical challenge of measuring millimetric differences between two imaging modalities with inherently different spatial resolution and tissue contrast properties, we acknowledge that a degree of inter-modality measurement variability is unavoidable. In our study, MRI tumor size was measured as the longest diameter of enhancing tumor on DCE sequences, while PET/CT tumor size was derived from the CT component using the longest axial diameter. Both measurements were performed by experienced readers following standardized criteria, and the requirement for same-center imaging at both time points ensured consistency of acquisition parameters within each modality. While individual millimetric differences should be interpreted with caution, the Cox regression analysis treats discordance as a continuous variable across the entire cohort, and the statistically significant association with survival outcomes suggests that the observed signal exceeds random measurement noise. Nevertheless, prospective validation with standardized, protocol-driven measurement approaches is essential to confirm these findings.

From a quantitative perspective, the magnitude of the effect—a 3% increase in mortality risk and a 1% increase in progression risk per millimeter of discordance—may appear modest at the individual patient level. However, in clinical practice, discordance values can range widely; in our cohort, the observed range spanned from 0 to over 50 mm, implying that patients at the upper end of the discordance spectrum may face substantially elevated cumulative risk. Moreover, the fact that this association was independent of pCR status underscores its potential as a complementary risk marker that captures biological information not reflected by conventional pathological evaluation. This is clinically meaningful because current post-NACT risk stratification relies predominantly on pathological response and molecular subtype, and the addition of an imaging-derived prognostic variable could refine patient selection for adjuvant therapy intensification.

It is also worth noting that the concept of inter-modality discordance aligns with the broader paradigm of multiparametric imaging in oncology, where the integration of complementary data streams—anatomic, metabolic, and functional—may yield a more comprehensive characterization of tumor biology than any single modality alone. In the neoadjuvant breast cancer setting, this principle is supported by emerging data suggesting that radiomics-based and artificial intelligence-driven multimodal integration may further improve predictive accuracy, although such approaches were beyond the scope of the present study.

If independently confirmed, the clinical implications of these observations may be substantial. Specifically, the independent prognostic value of dual-modality discordance suggests that integrated functional–anatomic assessment may identify high-risk populations requiring intensified surveillance, adjuvant therapy escalation, or clinical trial enrollment evaluating novel therapeutic strategies for high-risk residual disease. This finding is particularly relevant given that the achievement of pCR, while serving as an acceptable surrogate endpoint for survival outcomes [30], was not attained by 58.8% of patients in our series, with substantial outcome heterogeneity among those with residual disease. Post-NACT imaging discordance may therefore function as an orthogonal risk stratification tool, identifying patients whose residual disease characteristics confer elevated risk beyond that predicted by residual cancer burden score or molecular subtype alone.

With regard to the cost-effectiveness of dual-modality imaging, we acknowledge that the routine use of both MRI and ^18^F-FDG PET/CT for post-NACT response assessment represents a significant resource investment and is not current standard practice at most institutions, where PET/CT is typically reserved for staging purposes and MRI serves as the primary tool for local response evaluation. Our data do not allow for a formal cost-effectiveness analysis. However, the finding that MRI-PET discordance provides independent prognostic information beyond pCR—information that cannot be captured by either modality alone—suggests that there may be a subset of patients in whom dual-modality assessment offers clinically actionable risk stratification. We speculate that such an approach might be most cost-effective when applied selectively to patients with discordant or equivocal findings on the primary imaging modality, rather than universally. Prospective studies incorporating formal health economic analyses are needed to determine whether the prognostic benefit of dual-modality imaging justifies its incremental cost in specific clinical scenarios.

When compared with the existing literature, our findings both corroborate and extend prior observations. The strong correlation between post-NACT imaging response and pCR is consistent with the meta-analysis by Li et al. [7], which reported pooled sensitivity and specificity values of 0.65 and 0.88 for MRI and 0.86 and 0.72 for PET/CT, respectively. However, our study distinguishes itself in two important respects. First, while most prior investigations assessed MRI and PET/CT independently or in direct comparison, we specifically evaluated the prognostic significance of the discordance between the two modalities—a metric that has received limited attention in the literature. Second, our subtype-stratified analysis reveals that the predictive value of baseline imaging is confined to hormone receptor-positive HER2-negative disease, a finding that contrasts with studies reporting broader applicability of baseline tumor size as a predictor of pCR across subtypes [19,20]. This discrepancy likely reflects differences in cohort composition, treatment protocols, and pCR definitions. In particular, the high proportion of HER2-positive patients receiving dual-targeted therapy in our cohort may have attenuated the influence of tumor size on pCR in this subgroup, given the potent biological effect of anti-HER2 agents irrespective of tumor volume. These observations highlight the importance of molecular subtype as a critical confounder that must be accounted for in future imaging biomarker studies.

Several limitations warrant consideration in interpreting these findings. First, the retrospective design and reliance on clinical imaging reports may introduce potential measurement variability that standardized protocols would mitigate. In particular, PET/CT acquisition and reconstruction parameters (time per bed position, matrix size, number of OSEM iterations and subsets) varied across centers and over the study period; although the same-center requirement for paired examinations ensured within-patient consistency, between-patient variability in image quality cannot be excluded. Second, the cohort spans 2015–2025, during which NACT regimens evolved [31], though the most common regimens are in accordance with contemporary standards. The evolution of treatment protocols—particularly the introduction of dual HER2-targeted therapy and immune checkpoint inhibitors for triple-negative disease during the study period—may have influenced pCR rates differentially across temporal subgroups, and the retrospective design precluded a formal analysis of temporal trends in treatment response. Third, the median follow-up of 34.9 months limits survival data maturity, with event rates of 12.5% for progression and 12.2% for mortality, and longer follow-up is necessary to definitively establish the prognostic significance of imaging parameters, particularly in hormone receptor-positive disease where late recurrences are common. The low event rates may have limited the statistical power to detect smaller effect sizes in subgroup analyses, and the survival findings should be considered hypothesis-generating pending confirmation in cohorts with longer follow-up and more mature event data. Fourth, the study did not incorporate advanced techniques—including diffusion-weighted MRI, radiomics, or deep learning approaches—that may achieve superior pCR prediction. Fifth, the sample size within molecular subtype strata limited the statistical power for subgroup analyses. Sixth, although adjuvant treatments—including radiotherapy, endocrine therapy, and post-neoadjuvant systemic therapy escalation (T-DM1 for HER2-positive residual disease, capecitabine for triple-negative residual disease)—were administered in accordance with guideline-based institutional protocols as described in Section 2.5, detailed data on specific adjuvant regimens were not systematically collected, and their potential confounding effect on survival outcomes cannot be excluded. Seventh, data on the type of surgical procedure (breast-conserving surgery versus mastectomy) were not available for analysis; accordingly, the potential impact of surgical approach on disease-free survival and the interaction between surgical strategy, pCR achievement, and post-NACT imaging findings could not be assessed. Eighth, disease progression events were not classified by site (locoregional recurrence versus distant metastasis); this distinction would be important for interpreting the clinical significance of imaging discordance, particularly with respect to identifying predominant patterns of treatment failure. Future prospective studies should incorporate detailed adjuvant treatment data, surgical procedure type, and site-specific progression classification to address these gaps. Finally, the biological mechanisms underlying the association between imaging discordance and adverse outcomes remain speculative, requiring validation through correlative science investigations.

Despite these caveats, this study demonstrates that post-NACT MRI and ^18^F-FDG PET/CT show a strong correlation with pCR, whereas baseline MRI tumor size predicts pCR only in hormone receptor-positive, HER2-negative subtypes, emphasizing the pivotal role of molecular subtype in determining the performance of imaging biomarkers. The identification of post-NACT imaging discordance as an independent predictor of mortality and progression represents a novel finding suggesting that dual-modality assessment provides complementary prognostic information not captured by pathological evaluation alone. These findings support integrating functional and anatomic imaging for comprehensive response assessment and risk stratification, potentially identifying high-risk patients who may benefit from intensified surveillance or adjuvant therapy escalation. Prospective validation with standardized imaging protocols and longer follow-up is warranted to confirm these observations and establish their role in treatment decision-making.

## Figures and Tables

**Table 1:** Baseline characteristics of the study patients (*n* = 335).

Variable	Value
Age (years), mean ± SD	50.48 ± 10.59
Body mass index (kg/m^2^), mean ± SD	29.04 ± 5.52
Menopausal status, *n* (%)	
Premenopausal	144 (43.0%)
Perimenopausal	72 (21.5%)
Postmenopausal	119 (35.5%)
Clinical stage, *n* (%)	
Stage I	2 (0.6%)
Stage II	209 (62.4%)
Stage III	124 (37.0%)
Clinical T stage, *n* (%)	
T1	79 (23.6%)
T2	221 (66.0%)
T3	30 (9.0%)
T4	5 (1.5%)
Histological subtype, *n* (%)	
Invasive ductal carcinoma	238 (71.3%)
Invasive lobular carcinoma	9 (2.7%)
Mixed/Other	88 (26.3%)
Estrogen receptor status, *n* (%)	
Positive	187 (55.8%)
Weakly positive	45 (13.4%)
Negative	103 (30.7%)
HER2 status, *n* (%)	
Positive	199 (59.4%)
Negative	136 (40.6%)
Ki-67 (%), mean ± SD	41.99 ± 25.17
Ki-67 category, *n* (%)	
<20%	58 (17.3%)
≥20%	277 (82.7%)
Molecular subtype, *n* (%)	
Luminal A	23 (6.9%)
Luminal B HER2-negative	74 (22.1%)
Luminal B HER2-positive	136 (40.6%)
HER2-enriched	62 (18.5%)
Triple-negative	40 (11.9%)
Baseline imaging measurements, mean ± SD	
Baseline MRI tumor size (mm)	32.64 ± 19.39
Baseline ^18^F-FDG PET/CT tumor size (mm)	30.92 ± 16.02
Baseline ^18^F-FDG PET/CT SUVmax	10.30 ± 6.05
Presence of an in situ component, *n* (%)	142 (42.5%)
NACT regimen, *n* (%)	
4AC–taxane–trastuzumab–pertuzumab	136 (40.6%)
4AC–taxane	121 (36.1%)
Taxane–trastuzumab–pertuzumab	62 (18.5%)
Other regimens	16 (4.8%)
Number of chemotherapy cycles, mean ± SD	11.6 ± 4.5

Abbreviations: SD, standard deviation; HER2, human epidermal growth factor receptor 2; MRI, magnetic resonance imaging; ^18^F-FDG PET/CT, ^18^F-fluorodeoxyglucose positron emission tomography/computed tomography; SUVmax, maximum standardized uptake value; NACT, neoadjuvant chemotherapy; AC, doxorubicin (adriamycin) plus cyclophosphamide.

**Table 2:** Distribution of post-NACT imaging findings according to Miller–Payne pathological grade.

Miller–Payne Grade	1 (*n* = 20)	2 (*n* = 36)	3 (*n* = 79)	4 (*n* = 47)	5/pCR (*n* = 138)	*p*
Post-NACT ^18^F-FDG PET/CT response						<0.001
Complete response	0 (0%)	7 (19.4%)	30 (38.0%)	30 (63.8%)	115 (83.3%)	
Partial response	9 (45.0%)	27 (75.0%)	45 (57.0%)	17 (36.2%)	21 (15.2%)	
Stable/progressive disease	9 (45.0%)	1 (2.8%)	3 (3.8%)	0 (0%)	1 (0.7%)	
Missing data	2 (10.0%)	1 (2.8%)	1 (1.3%)	0 (0%)	1 (0.7%)	
Post-NACT MRI response						<0.001
Complete response	1 (5.0%)	1 (2.8%)	8 (10.1%)	11 (23.4%)	110 (79.7%)	
Partial response	8 (40.0%)	31 (86.1%)	63 (79.7%)	35 (74.5%)	24 (17.4%)	
Stable/progressive disease	8 (40.0%)	3 (8.3%)	4 (5.1%)	0 (0%)	0 (0%)	
Missing data	3 (15.0%)	1 (2.8%)	4 (5.1%)	1 (2.1%)	4 (2.9%)	
Post-NACT ^18^F-FDG PET/CT tumor size (mm)	38.00 ± 22.45	18.66 ± 9.13	21.39 ± 13.65	12.93 ± 5.14	16.09 ± 7.71	<0.001
Post-NACT MRI tumor size (mm)	40.31 ± 16.20	24.03 ± 15.51	22.53 ± 14.09	9.94 ± 4.32	16.60 ± 10.30	<0.001
Post-NACT ^18^F-FDG PET/CT SUVmax	6.15 ± 5.89	3.12 ± 4.36	2.84 ± 5.52	0.96 ± 1.85	0.45 ± 1.33	<0.001
^18^F-FDG PET/CT metabolic change (ΔFDG)	3.85 ± 6.42	6.88 ± 6.64	7.46 ± 6.95	9.04 ± 5.32	9.67 ± 5.50	<0.001

Abbreviations: pCR, pathologic complete response; NACT, neoadjuvant chemotherapy; MRI, magnetic resonance imaging; ^18^F-FDG PET/CT, ^18^F-fluorodeoxyglucose positron emission tomography/computed tomography; SUVmax, maximum standardized uptake value; FDG, fluorodeoxyglucose.

**Table 3:** Predictive Performance of Baseline MRI and 18F-FDG PET/CT Parameters for pCR by Molecular Subtype.

Molecular Breast Cancer Subtype	Baseline Imaging Parameter	AUC (95% CI)	*p*-Value	Optimal Cut-Off	Sensitivity (%)	Specificity (%)
Luminal A + Luminal B HER2-negative (*n* = 97)						
	MRI tumor size (mm)	0.681 (0.558–0.804)	0.013	≤28.5	60.0	62.2
	^18^F-FDG PET/CT tumor size (mm)	0.654 (0.527–0.781)	0.039	≤26.0	57.9	56.8
	^18^F-FDG PET/CT SUVmax	0.612 (0.484–0.740)	0.130	≤9.5	63.2	59.5
Luminal B HER2+ + HER2-enriched + Triple-negative (*n* = 238)						
	MRI tumor size (mm)	0.528 (0.453–0.603)	0.471	≤28.5	52.8	52.0
	^18^F-FDG PET/CT tumor size (mm)	0.565 (0.491–0.639)	0.091	≤25.5	54.6	54.9
	^18^F-FDG PET/CT SUVmax	0.533 (0.458–0.608)	0.411	≤8.5	51.4	53.1

Abbreviations: HER2. human epidermal growth factor receptor 2; ^18^F-FDG PET/CT, ^18^F-fluorodeoxyglucose positron emission tomography/computed tomography; SUVmax, maximum standardized uptake value; AUC, area under the receiver operating characteristic curve; CI, confidence interval.

**Table 4:** Multivariable Cox regression analysis of factors associated with survival outcomes.

Variable	Overall Survival		Progression-Free Survival	
	HR (95% CI)	*p*	HR (95% CI)	*p*
pCR (yes versus no)	0.34 (0.10–0.94)	0.008	0.40 (0.19–0.86)	0.019
MRI-PET discordance (per mm)	1.03 (1.01–1.15)	0.005	1.01 (1.00–1.03)	0.049
PET metabolic change—ΔFDG (per unit)	—	ns	0.95 (0.90–1.01)	0.126
Age group (versus 18–30 years)	—	—		0.065
31–40	—	—	0.41 (0.12–1.41)	0.157
41–50	—	—	0.26 (0.08–0.81)	0.021
51–60	—	—	0.10 (0.02–0.39)	0.001
>60	—	—	0.18 (0.04–0.66)	0.010

Abbreviations: OS, overall survival; PFS, progression-free survival; pCR, pathologic complete response; MRI, magnetic resonance imaging; PET, positron emission tomography; FDG, fluorodeoxyglucose; ΔFDG, change in fluorodeoxyglucose uptake; HR, hazard ratio; CI, confidence interval; ns, not significant.

## Data Availability

The data supporting the findings of this study are available within the article. Any additional data requests or inquiries regarding the primary datasets should be directed to the corresponding author.

## Abbreviations

The following abbreviations are used in this manuscript:

BC	Breast cancer
NACT	Neoadjuvant chemotherapy
pCR	Pathological complete response
MRI	Magnetic resonance imaging
^18^F-FDG PET/CT	^18^F-fluorodeoxyglucose positron emission tomography/computed tomography
HR	Hormone receptor
HER2	Human epidermal growth factor receptor 2
SUVmax	Maximum standardized uptake value
ΔFDG	Metabolic change (change in fluorodeoxyglucose uptake)
ER	Estrogen receptor
PR	Progesterone receptor
OS	Overall survival
PFS	Progression-free survival
BCS	Breast-conserving surgery
BMI	Body mass index
SD	Standard deviation
AC	Doxorubicin (adriamycin) plus cyclophosphamide
ANOVA	Analysis of variance
OR	Odds ratio
CI	Confidence interval
ROC	Receiver operating characteristic
AUC	Area under the receiver operating characteristic curve
HR	Hazard ratio
NS	Not significant
PET	Positron emission tomography
FDG	Fluorodeoxyglucose
T-DM1	Trastuzumab emtansine
OSEM	Ordered-subset expectation maximization
EPV	Events per variable

## References

[1] Chen Y., Qi Y., Wang K. (2023). Neoadjuvant chemotherapy for breast cancer: An evaluation of its efficacy and research progress. Front. Oncol..

[2] Spring L.M., Fell G., Arfe A., Sharma C., Greenup R., Reynolds K.L., Smith B.L., Alexander B., Moy B., Isakoff S.J. (2020). Pathologic Complete Response after Neoadjuvant Chemotherapy and Impact on Breast Cancer Recurrence and Survival: A Comprehensive Meta-analysis. Clin. Cancer Res..

[3] Pennisi A., Kieber-Emmons T., Makhoul I., Hutchins L. (2016). Relevance of pathological complete response after neoadjuvant therapy for breast cancer. Breast Cancer.

[4] Kong X., Zhang Q., Wu X., Zou T., Duan J., Song S., Nie J., Tao C., Tang M., Wang M. (2022). Advances in imaging in evaluating the efficacy of neoadjuvant chemotherapy for breast cancer. Front. Oncol..

[5] AlBuainain R.Y., Bunajem F.Y., Abdulla H.A. (2025). Assessment of tumor response to neoadjuvant chemotherapy in breast cancer using MRI and 18F-FDG PET/CT. Eur. J. Breast Health.

[6] Alshaibani N., Chandramohan J.K., Althawadi Y., Almusalam M., Khairi S.S., Saif H.S., Al Sindi K., Aly S. (2024). Accuracy of MRI versus PET/CT in the prediction of treatment response to neoadjuvant chemotherapy in breast cancer. Cureus.

[7] Li H., Yao L., Jin P., Hu L., Li X., Guo T., Yang K. (2018). MRI and PET/CT for evaluation of the pathological response to neoadjuvant chemotherapy in breast cancer: A systematic review and meta-analysis. Breast.

[8] Reig B., Lewin A.A., Du L., Heacock L., Toth H.K., Heller S.L., Gao Y., Moy L. (2021). Breast MRI for evaluation of response to neoadjuvant therapy. Radiographics.

[9] Gelardi F., Tiberio P., Torrisi R., Zanca R., Rodari M., Zambelli A., Santoro A., Fernandes B., Sagona A., Errico V. (2025). Using [18F]FDG PET/CT to identify optimal responders to neoadjuvant therapy in breast cancer-results from a prospective patient cohort. Cancers.

[10] Ghezzawi M., El Charif M.H., Lteif L., Panossian V., Haddad A., Sarkis S., Zebian Y., Kheil M., Maktabi M.A., Fakhruddin N. (2025). Patterns of response of breast cancer after neoadjuvant chemotherapy according to molecular subtype. Front. Oncol..

[11] Kim S.I., Sohn J., Koo J.S., Park S.H., Park H.S., Park B.W. (2010). Molecular subtypes and tumor response to neoadjuvant chemotherapy in patients with locally advanced breast cancer. Oncology.

[12] Jeh S.K., Kim S.H., Kang B.J. (2013). Comparison of the diagnostic performance of response evaluation criteria in solid tumor 1.0 with response evaluation criteria in solid tumor 1.1 on MRI in advanced breast cancer response evaluation to neoadjuvant chemotherapy. Korean J. Radiol..

[13] Can C., Akdeniz N., Kömek H., Gündoğan C., Urakçı Z., Işıkdoğan A. (2022). The prognostic role of baseline 18F-FDG PET/CT SUVmax and SUVmax change in patients with node-positive breast cancer receiving neoadjuvant chemotherapy. Rev. Esp. Med. Nucl. Imagen Mol..

[14] An Y.Y., Kim S.H., Kang B.J., Lee A.W. (2015). Treatment response evaluation of breast cancer after neoadjuvant chemotherapy and usefulness of the imaging parameters of MRI and PET/CT. J. Korean Med. Sci..

[15] Cosar R., Sut N., Ozen A., Tastekin E., Topaloglu S., Cicin I., Nurlu D., Ozler T., Demir S., Yıldız G. (2022). Breast cancer subtypes and prognosis: Answers to subgroup classification questions, identifying the worst subgroup in our single-center series. Breast Cancer.

[16] Ma J., Deng Y., Chen D., Li X., Yu Z., Wang H., Zhong L., Li Y., Wang C., Li X. (2023). Spatial immunophenotypes orchestrate prognosis in triple-negative breast cancer with Miller-Payne grade 4 following neoadjuvant chemotherapy. NPJ Breast Cancer.

[17] McFarland D.C., Naikan J., Rozenblit M., Mandeli J., Bleiweiss I., Tiersten A. (2016). Changes in pathological complete response rates after neoadjuvant chemotherapy for breast carcinoma over five years. J. Oncol..

[18] Bischoff H., Espié M., Petit T. (2024). Neoadjuvant therapy: Current landscape and future horizons for ER-positive/HER2-negative and triple-negative early breast cancer. Curr. Treat. Options Oncol..

[19] Prati R., Minami C.A., Gornbein J.A., Debruhl N., Chung D., Chang H.R. (2009). Accuracy of clinical evaluation of locally advanced breast cancer in patients receiving neoadjuvant chemotherapy. Cancer.

[20] Narui K., Ishikawa T., Oba M.S., Hasegawa Y., Kaise H., Kawate T., Yamada A., Yamada K., Suzuki Y., Niikura N. (2020). Prediction of pathological complete response after neoadjuvant chemotherapy in breast cancer by combining magnetic resonance imaging and core needle biopsy. Surg. Oncol..

[21] Dose-Schwarz J., Tiling R., Avril-Sassen S., Mahner S., Lebeau A., Weber C., Schwaiger M., Jänicke F., Untch M., Avril N. (2010). Assessment of residual tumour by FDG-PET: Conventional imaging and clinical examination following primary chemotherapy of large and locally advanced breast cancer. Br. J. Cancer.

[22] Eby P.R., Partridge S.C., White S.W., Doot R.K., Dunnwald L.K., Schubert E.K., Kurland B.F., Lehman C.D., Mankoff D.A. (2008). Metabolic and vascular features of dynamic contrast-enhanced breast magnetic resonance imaging and (15)O-water positron emission tomography blood flow in breast cancer. Acad. Radiol..

[23] Liu C., Sun L., Niu N., Hou P., Chen G., Wang H., Zhang Z., Jiang X., Xu Q., Zhao Y. (2025). Molecular classification of hormone receptor-positive/HER2-positive breast cancer reveals potential neoadjuvant therapeutic strategies. Signal Transduct. Target. Ther..

[24] Turner K.M., Yeo S.K., Holm T.M., Shaughnessy E., Guan J.L. (2021). Heterogeneity within molecular subtypes of breast cancer. Am. J. Physiol. Cell Physiol..

[25] Sekoba N., Demetriou D., Chauke-Malinga N., Mabeta P. (2025). Emerging strategies for targeting vasculogenic mimicry in breast cancer treatment. Discov. Oncol..

[26] Mo Z., Yang M., Zhu Z., Wang M., Wang H., Zhang Z., Lyu S., Xu F., Shang H., Lin H. (2025). Multiomic integration reveals subtype-specific predictors of neoadjuvant treatment response in breast cancer. Sci. Adv..

[27] Allott E.H., Geradts J., Sun X., Cohen S.M., Zirpoli G.R., Khoury T., Bshara W., Chen M., Sherman M.E., Palmer J.R. (2016). Intratumoral heterogeneity as a source of discordance in breast cancer biomarker classification. Breast Cancer Res..

[28] Kim J.A., Son E.J., Youk J.H., Kim E.K., Kim M.J., Kwak J.Y., Jeong J. (2011). MRI findings of pure ductal carcinoma in situ: Kinetic characteristics compared according to lesion type and histopathologic factors. AJR Am. J. Roentgenol..

[29] Groheux D., Vaz S.C., Poortmans P., Mann R.M., Ulaner G.A., Cook G.J.R., Hindié E., Pilkington Woll J.P., Jacene H., Rubio I.T. (2024). Role of [18F]FDG PET/CT in patients with invasive breast carcinoma of no special type: Literature review and comparison between guidelines. Breast.

[30] Conforti F., Pala L., Sala I., Oriecuia C., De Pas T., Specchia C., Graffeo R., Pagan E., Queirolo P., Pennacchioli E. (2021). Evaluation of pathological complete response as surrogate endpoint in neoadjuvant randomised clinical trials of early stage breast cancer: Systematic review and meta-analysis. BMJ.

[31] Li Z., Wang Y. (2024). Evolution of neoadjuvant therapy for breast cancer regimens over 12 years and pathologic response rates according to tumor subtypes and clinical stage: A single-center retrospective study. J. Cancer Res. Ther..

